# The Long Noncoding RNA *IFNG-AS1* Promotes T Helper Type 1 Cells Response in Patients with Hashimoto’s Thyroiditis

**DOI:** 10.1038/srep17702

**Published:** 2015-12-04

**Authors:** Huiyong Peng, Yingzhao Liu, Jie Tian, Jie Ma, Xinyi Tang, Ke Rui, Xinyu Tian, Chaoming Mao, Liwei Lu, Huaxi Xu, Pengcheng Jiang, Shengjun Wang

**Affiliations:** 1 Department of Laboratory Medicine, The Affiliated People’s Hospital, Jiangsu University, Zhenjiang, 212002, China; 2 Institute of Laboratory Medicine, Jiangsu Key Laboratory of Laboratory Medicine, Jiangsu University School of Medicine, Zhenjiang, 212013, China; 3 Department of Endocrinology, The Affiliated People’s Hospital, Jiangsu University, Zhenjiang, 212002, China; 4 Department of Pathology, The University of Hong Kong, Hong Kong, China; 5 Department of General Surgery, The Affiliated People’s Hospital, Jiangsu University, Zhenjiang, 212002, China

## Abstract

The long noncoding (lnc) RNA-*Ifng-AS1* plays an essential role in the transcription of the gene encoding IFN-γ by Th1 cells, and its human ortholog, *IFNG-AS1*, is expressed in human Th1 cells. However, *IFNG-AS1* contributing to Th1 cells’ response in Hashimoto’s thyroiditis (HT) patients has not been reported. Twenty-eight HT patients and 20 healthy controls were enrolled in the study. The proportion of circulating Th1 cells and the level of T-bet, *IFNG* mRNA were increased in HT patients, the expression of *IFNG-AS1* was upregulated and positively correlated with the proportion of circulating Th1 cells or T-bet, and *IFNG* expression, or serum level of anti-thyroglobulin antibody/thyroperoxidase antibody in HT patients. *IFNG-AS1* regulated the expression of *IFNG* at both transcriptional and translational level in human CD4^+^ T cells. Furthermore, strong positive correlations between the increased transcript level of *IFNG-AS1* and the increased transcript level of T-bet or *IFNG* were revealed in thyroid tissues from HT patients. Our results indicate that enhanced expression of lncRNA-*IFNG-AS1* contributes to Th1 cell response in HT patients and may be involved in the pathogenesis of HT.

Hashimoto’s thyroiditis (HT), also named chronic lymphocytic thyroiditis, is a clinical, organ-specific autoimmune disease characterized by lymphocytic infiltration of the thyroid parenchyma, diffusely enlarged thyroid gland and elevated production of autoantibodies, mainly including anti-thyroglobulin antibody (TgAb) and thyroperoxidase antibody (TPOAb)^1^,^2^. It is now considered to be the most common autoimmune disease and is often associated with other autoimmune diseases, such as Graves’ disease, systemic lupus erythematosus and rheumatoid arthritis^3^,^4^,^5^. HT is a complex and ever-expanding disease caused by genetic susceptibility, environmental factors and immunopathogenesis^6^. Accumulated evidence over recent years has suggested that CD4^+^ T helper cells disorder might be involved in the pathogenesis of HT^7^,^8^.

CD4^+^ T helper cells can be classified into Th1, Th2, Th17 and follicular helper T cells, based on the cytokine production^9^. The Th1 cell lineage is an inflammatory CD4^+^ T cells subset that mainly produces interferon gamma (IFN-γ), an inflammatory cytokine that participates protectively against intracellular microbes, delayed type hypersensitivity and autoimmune diseases^10^,^11^. The development and differentiation of Th1 cells depends on the activation of JAK/STAT pathway components STAT1 and STAT4 in the presence of interleukin 12^12^,^13^. T-bet is the key transcription factor, contributing to the transcription of *IFNG* in Th1 cells^14^. Recent studies have shown that Th1 cells might be involved in the development of HT^7^,^15^,^16^. However, our understanding of increased Th1 cells in HT patients remains largely unknown.

Long noncoding RNAs (lncRNAs) are increasingly appreciated as key regulators of genome expression, and only some of their functions have been characterized^17^. In some well-studied cases, lncRNAs play critical roles in various biological processes and diseases^18^,^19^,^20^. However, only a few lncRNAs, such as *lnc-DC*, *NRON*, *Gas5*, have been described in the regulation of the immune system by regulating particular genes^21^,^22^,^23^. *Ifng-AS1* (*Ifng* antisense RNA 1), also named *NeST* (nettoie Salmonella pas Theiler’s), or *Tmevpg1* (Theiler’s murine encephalomyelitis virus persistence candidate gene 1), a lncRNA was initially identified as a candidate gene for the control of Theiler’s virus persistence^24^. *Ifng-AS1* and its human ortholog are located adjacent to IFN-γ-encoding gene in both mouse and human. Recent studies have described that *Tmevpg1* (*Ifng-AS1, NeST*) is recognized to be a key checkpoint that requires T-bet for active transcription and contributes to *Ifng* expression in Th1 cells^25^,^26^. However, it is not yet known whether *IFNG-AS1* regulates Th1 cells response in HT patients. Therefore, we explore the role of *IFNG-AS1* in the pathogenesis of HT.

In this study, we investigated whether the expression of *IFNG-AS1* is dysregulated in HT patients. We found that the expression of *IFNG-AS1* was upregulated, and positively correlated with the proportion of circulating Th1 cells in HT patients. These findings provide new insights in understanding the role of *IFNG-AS1* in the pathogenesis of HT.

## Materials and Methods

### Subjects and samples

Twenty-eight patients with HT, including twenty-three females and five males were enrolled into the study. The main clinical characteristics of these patients are summarized in Table 1. All patients were diagnosed by clinical manifestation and auxiliary examination, including B-ultrasonic and laboratory criteria. The serum concentration of free triiodothyronine (FT3), free thyroxine (FT4), thyroid stimulating hormone (TSH), TgAb, and TPOAb were measured by LDX-800 (BECKMAN COULTER, California, USA), according to the manufacturer’s instructions. Ten HT patients with hypothyroidism had a high level of TSH and low level of FT4, other patients with euthyroid had a normal level of both TSH and FT4. All patients had a positive test for TgAb and TPOAb. Twenty age- and sex-matched healthy subjects were included as controls. All healthy subjects were free of thyroid-specific autoantibodies and had no history of thyroid disease or other autoimmune diseases. The number of peripheral leukocytes was within normal range. Peripheral blood samples were obtained from all patients and healthy controls.

Fresh tissue samples from the thyroid gland of ten HT patients were collected from thyroidectomy and stored at −80 °C. Lymphocytic infiltration was detected in thyroid samples. Thyroid tissues from five patients with simple goiter were used as control thyroid samples.

The study conformed to the principles outlined in the Declaration of Helsinki and in accordance with the approved guidelines. Written informed consent was obtained from each participant prior to blood samples collection. All samples were taken in accordance with the regulations and approval of the Affiliated People’s Hospital of Jiangsu University.

### Cell isolation and purification

Human peripheral blood mononuclear cells (PBMCs) were isolated by density-gradient centrifugation over Ficoll-Hypaque solution (Haoyang Biological Technology Co., Tianjin, China) and stored at −80 °C until use for quantitative real-time PCR (qRT-PCR). Human CD4^+^ T cells were purified from PBMCs by magnetic beads using CD4^+^ T cell Isolation Kit (Miltenyi Biotec GmbH, Bergisch Gladbach, Germany) according to the manufacturer’s instructions. Human CD4^+^ T cells were cultured in RPMI-1640 medium (Gibco, California, USA) supplemented with 10% fetal bovine serum (Gibco, California, USA) for transfection.

Thyroid mononuclear cells (TMCs) were obtained from thyroid specimens, which were minced and digested with collagenase II (Sigma-Aldrich, St. Louis, MO) for 1–2 h at 37 °C and then isolated by density-gradient centrifugation over Ficoll-Hypaque solution. Cell viability was found to be more than 95%.

### Flow cytometric analysis

Separated PBMCs were resuspended at 1 × 10^6^/ml in RPMI-1640 medium containing 10% fetal bovine serum and stimulated with 50 ng/ml of phorbol myristate acetate (PMA; Sigma-Aldrich, California, USA) and 1 μg/ml of ionomycin (Sigma-Aldrich, California, USA) for 2 hours and then incubated for an additional 4 hours in the presence of 1 μg/ml of brefeldin-A (eBioscience, San Diego, USA) at 37 °C and 5% CO_2_. After the incubation, the suspended cells were stained with phycoerythrin-cyanin 5 (PE-Cy5) -conjugated anti-human CD3 mAb and fluorescein isothiocyanate (FITC) -conjugated anti-human CD8 mAb (eBioscience, San Diego, USA) against cell surface antigens for thirty minutes at 4 °C in the dark. Cells were then fixed and permeabilized using an intracellular staining kit (Invitrogen, Carlsbad, USA), followed by incubation for 45 minutes at 4 °C in the dark with PE-conjugated anti-human IFN-γ mAb (Miltenyi Biotec GmbH, Bergisch Gladbach, Germany). The stained cells were analyzed with Accuri C6 (Becton Dickinson, San Jose, USA). To analyze the proportion of Th1 cells, the population of CD3^+^ CD8^-^ IFN-γ^+^ cells was defined as Th1 cells.

### RNA Isolation and Quantitative Real-Time PCR

Total RNA was isolated from PBMCs with TRIzol reagent (Invitrogen, California, USA) according to the manufacturer’s instructions. The cDNA was synthesized with random primer and ReverTraAca^®^qPCR RT kit (Toyobo, Osaka, Japan). Quantitative real-time PCR was performed in triplicate using Bio-Rad SYBR green super mix (Bio-Rad, Hercules, USA). Primer sequences were as follows: T-bet, sense, 5′-TTGAGGTGAACGACGGAGAG-3′, antisense, 5′-GGCATTCTGGTAGGCAGTCA-3′; *IFNG*, sense, 5′-GAGTGTGGAGACCATCAAGGA-3′, antisense, 5′-TGTATTGCTTTGCGTTGGAC-3′; *IFNG-AS1*, sense, 5′-GCTGATGATGGTGGTGGCAATCT-3′, antisense, 5′-TTAGCAGTTGGTGGGCTTCT-3′. *β-actin*, sense, 5′-GAGTGTGGAGACCATCAAGGA-3′, antisense, 5′-TGTATTGCTTTGCGTTGGAC-3′. The level of each gene was expressed as the ratio to *β-actin* transcript level. Data were analyzed with Bio-Rad CFX Manager software.

### Small interfering RNA knockdown

Small interfering RNA (siRNA) (Ribobio, Guangzhou, China) was designed against the sequence of *IFNG-AS1*. Nonspecific scramble siRNA was used as negative control (NC). The purified human CD4^+^ T cells were transfected with the *IFNG-AS1* siRNA or NC at 100 nM dose using the Entranster-R (Engreen Biosystem, Co Ltd, Beijing, China) according to the manufacturers’ instructions for 48 hours in the presence of 0.5 μg/ml functional anti-human CD3 mAb plus 2 μg/ml functional anti-human CD28 mAb (Miltenyi Biotec GmbH, Bergisch Gladbach, Germany). IFN-γ^+^ cells were measured by flow cytometric analysis.

### Statistical analysis

Student’s unpaired *t* test was applied for two comparisons in accordance with the standard *t* test. Mann-Whitney *U* test was used to analyze the difference between the two groups. Correlation between variables was determined by Pearson’s correlation coefficient. *p* value < 0.05 was considerate as significant (*p < 0.05, **p < 0.01, ***p < 0.001). Data were analyzed with GrapPadPrism version 5 software (GraphPad Software, Inc., San Diego, USA).

## Results

### Increased circulating Th1 cells in HT patients

**Figure 1:**
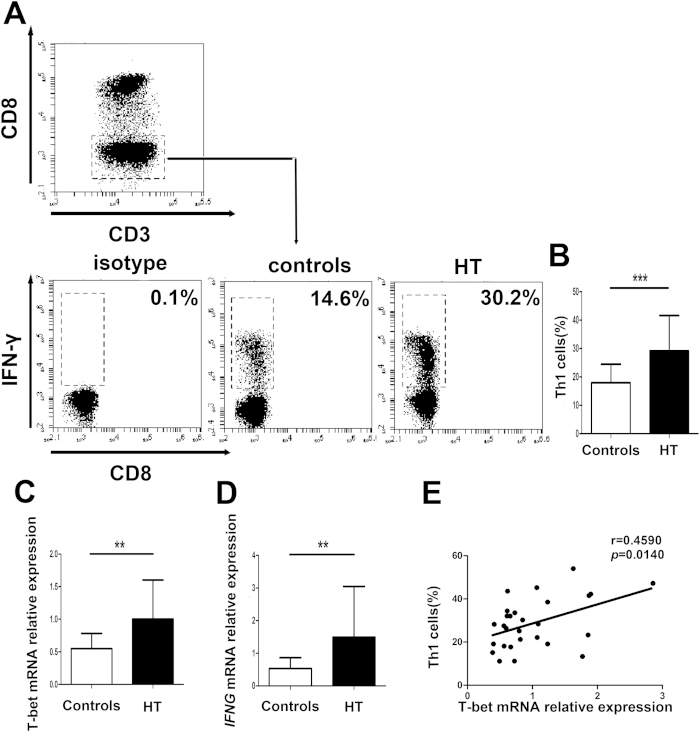
Increased circulating Th1 cells in peripheral blood from HT patients. Peripheral blood was obtained from 28 HT patients and 20 healthy controls. (**A**) Representative flow cytometry dot plots of Th1 cells in HT patients and healthy controls are shown. Values in the upper left rectangular region correspond to the proportion of Th1 cells. We used isotype control to determine the positive cells, and all of the values were gated on CD3^+^ CD8^-^ cells. (**B**) The percentages of Th1 cells were compared between HT patients and healthy controls. (**C**) The transcript level of T-bet mRNA in PBMCs from HT patients and healthy controls was determined by qRT-PCR. (**D**) The transcript level of *IFNG* mRNA in PBMCs from HT patients and healthy controls was determined by qRT-PCR. (**E**) The correlation between the transcript level of T-bet mRNA and the percentage of Th1 cells in peripheral blood from 28 HT patients. Each data point represents an individual subject, horizontal lines show the mean. *p < 0.05; **p < 0.01.

To quantify peripheral Th1 cells in patients with HT, we first gated on CD3^+^ CD8^-^ cells as CD4^+^ T cells owing to the downregulated expression of surface membrane CD4 molecule on PBMCs after being stimulated with PMA and ionomycin^27^ and then identified IFN-γ^+^ cells to distinguish Th1 cells from activated PBMCs (Fig. 1A). The proportion of peripheral Th1 cells in PBMCs from patients with HT was significantly higher than that in healthy controls (Fig. 1B).

Subsequently, we determined the transcript level of T-bet and *IFNG* in PBMCs from HT patients and healthy controls by qRT-PCR and found that the transcript level of T-bet and *IFNG* were substantially greater in HT patients than in healthy controls (Fig. 1C,D). Moreover, a positive correlation between the transcript level of T-bet and the proportion of Th1 cells was found in HT patients (r = 0.4590; *p* = 0.0140) (Fig. 1E).

### Positive correlations between elevated transcript level of *IFNG-AS1* and increased circulating Th1 cells in HT patients

**Figure 2:**
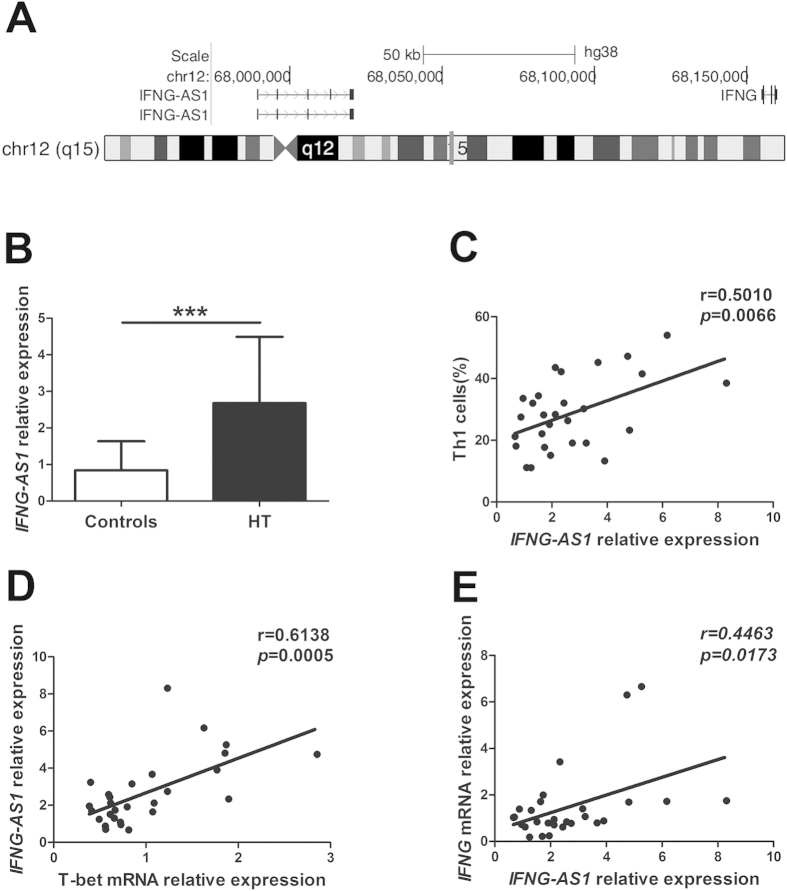
The correlation between the transcript level of *IFNG-AS1* and Th1 cells in HT patients. (**A**) Genomic position of *IFNG-AS1* on human chromosome. (**B**) The transcript level of *IFNG-AS1* in PBMCs from 28 HT patients and 20 healthy controls was determined by qRT-PCR. (**C**) The correlation between the transcript level of *IFNG-AS1* and the percentage of Th1 cells in 28 HT patients. (**D**) The correlation between the transcript level of *IFNG-AS1* and the transcript level of T-bet mRNA in 28 HT patients. (**E**) The correlation between the transcript level of *IFNG-AS1* and the transcript level of *IFNG* mRNA in 28 HT patients. Each data point represent an individual subject, horizontal lines show the mean. ***p < 0.001.

*IFNG-AS1* is a long noncoding RNA that is comprised of four exons and located at Chromosome 12q15 on the opposing strand to *IFNG* (Fig. 2A). *Ifng-AS1* (*Tmevpg1, NeST*) expression contributes to driving Th1 cell-dependent *Ifng* expression, and its human ortholog, *IFNG-AS1* (*TMEVPG1, NEST*), is selectively expressed in Th1 cells^25^. To address the possibility that *IFNG-AS1* contributes to increased Th1 cells in HT patients, the transcript level of *IFNG-AS1* was determined by qRT-PCR. As shown in Fig. 2B, the transcript level of *IFNG-AS1* from PBMCs was increased in HT patients compared with that in healthy controls. Moreover, positive correlations were observed between the transcript level of *IFNG-AS1* and the percentage of Th1 cells (r = 0.5010; *p* = 0.0066) (Fig. 2C) or the transcript level of T-bet (r = 0.6138; *p* = 0.0005) (Fig. 2D) or the transcript level of *IFNG* (r = 0.4463; *p* = 0.0173) in HT patients (Fig. 2E). In contrast to the relationship we observed between the transcript level of *IFNG-AS1* and the proportion of Th1 cells, there was no correlation between the transcript level of *IFNG-AS1* and the proportion of CD8^+^ IFN-γ^+^ T cells (Supplemental Fig. 1A), which was significantly greater in HT patients than in healthy controls (Supplemental Fig. 1B).

### Influence of *IFNG-AS1* on the transcription of *IFNG* in human CD4^+^ T cells

**Figure 3:**
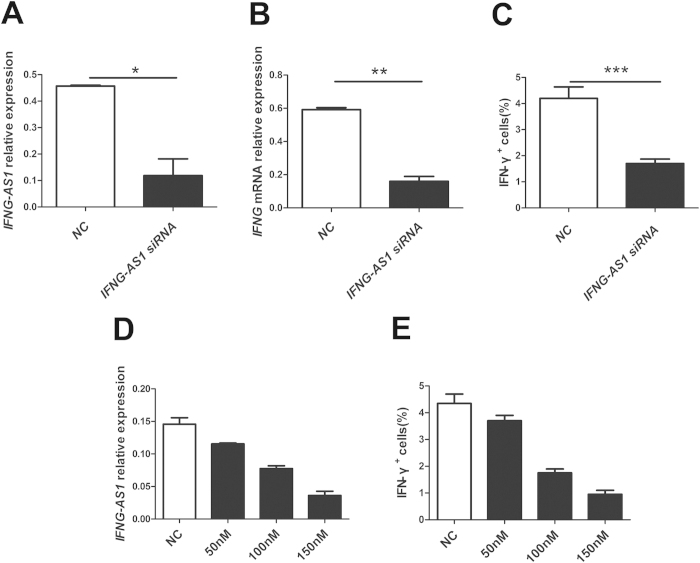
Influence of *IFNG-AS1* on the transcription of *IFNG in vitro*. Human CD4^+^ T cells were purified from PBMCs by magnetic beads and transfected with *IFNG-AS1* -specific siRNA and negative control (100 nM) in the presence of functional anti-human CD3 mAb and anti-human CD28 mAb for 24 h to determine the transcript level of *IFNG*, or 48 h before restimulation with PMA, ionomycin and brefeldin-A to determine the proportion of IFN-γ^+^ cells. (**A**) The transcript level of *IFNG-AS1* was determined by qRT-PCR after transfection. (**B**) The transcript level of *IFNG* mRNA was determined by qRT-PCR after transfection. (**C**) The proportion of IFN-γ^+^ cells was analyzed by flow cytometric analysis. (**D**) The transcript level of *IFNG-AS1* was detected by qRT-PCR after transfection after transfection with *IFNG-AS1*-specific siRNA in a dose-dependent manner. (**E**) The proportion of IFN-γ^+^ cells was analyzed by flow cytometric analysis after transfection with *IFNG-AS1*-specific siRNA in a dose-dependent manner. The results are indicated as the means ± SD of three independent experiments, horizontal lines show the mean. *p < 0.05; **p < 0.01; ***p < 0.001.

To determine whether *IFNG-AS1* affects *IFNG* transcription from CD4^+^ T cells, human purified CD4^+^ T cells were transfected with *IFNG-AS1*-specific siRNA and negative control. Manipulation of *IFNG-AS1*-specific siRNA resulted in the reduction of the transcript level of *IFNG-AS1* and *IFNG* compared with that of the negative control (Fig. 3A,B). Down-regulated expression of *IFNG-AS1* with siRNA resulted in the reduction of the percentage of IFN-γ^+^ cells compared with that of the negative control (Fig. 3C). Moreover, *IFNG-AS1*-specific siRNA suppressed the percentage of IFN-γ^+^ cells in a dose-dependent manner (Fig. 3D,E). Together, these results indicate that *IFNG-AS1* regulates the expression of *IFNG* in human CD4^+^ T cells.

### Increased expression of *IFNG-AS1* with elevated production of autoantibodies in HT patients

**Figure 4:**
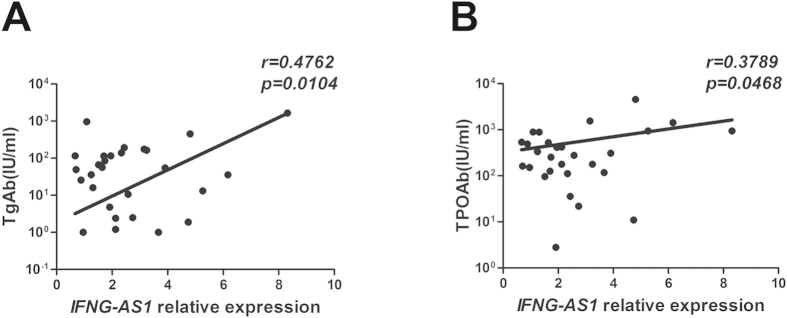
The correlation between the transcript level of *IFNG-AS1* and serum autoantibodies in HT patients. The correlation between the transcript level of *IFNG-AS1* and serum concentrations of TgAb (**A**) and TPOAb (**B**) in 28 HT patients.

TgAb and TPOAb participate in thyroid destruction in the manner of antibody-dependent cell-mediated cytotoxicity and activation of complement. These autoantibodies are a critical diagnosis index of HT^28^. Our results indicated that there were positive correlations between the transcript level of *IFNG-AS1* and the level of TgAb (r = 0.4762, *p* = 0.0104) or TPOAb (r = 0.3789, *p* = 0.0468) (Fig. 4A,B).

### Upregulated expression of *IFNG-AS1*, T-bet and *IFNG* mRNA in thyroid tissues from HT patients

**Figure 5:**
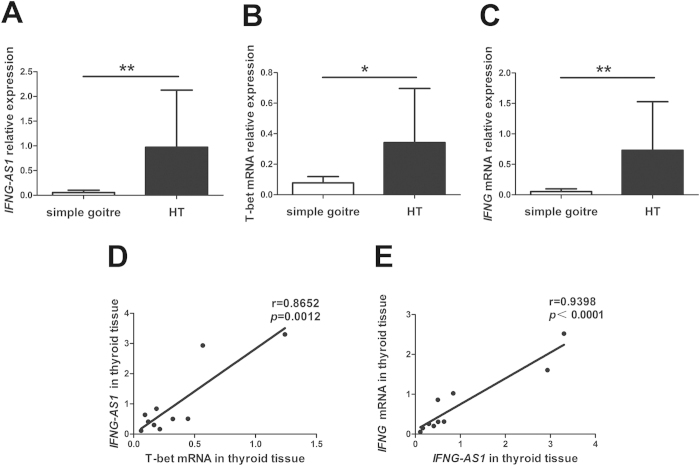
Upregulated expression of *IFNG-AS1*, T-bet and *IFNG* mRNA in thyroid tissues. The transcript level of *IFNG-AS1* (**A**), T-bet mRNA (**B**) and *IFNG* mRNA (**C**) in thyroid glands were determined by qRT-PCR from 10 patients with HT and 5 patients with simple goiter. Correlations between the transcript level of *IFNG-AS1* and T-bet mRNA (**D**) or *IFNG* mRNA (**E**) in thyroid glands from 10 HT patients. Each data point represents an individual subject, horizontal lines show the mean. *p < 0.05; **p < 0.01.

HT is an organ-specific autoimmune disease characterized by lymphoid infiltration and thyroid structure destruction. To determine whether *IFNG-AS1*, T-bet and *IFNG* mRNA were also expressed in local thyroid tissue, qRT-PCR analysis displayed enhanced expression of *IFNG-AS1*, T-bet and *IFNG* mRNA in TMCs from patients with HT compared to those from patients with simple goiter (Fig. 5A–C). Strong positive correlations were observed between the transcript level of *IFNG-AS1* and T-bet mRNA (r = 0.8652, *p* = 0.0012) or *IFNG* mRNA (r = 0.9398, *p* *<* 0.0001) in patients with HT (Fig. 5D,E).

## Discussion

Lower conservative expression of lncRNAs sequences has prevented most of the sequences from being functionally characterized^29^. Presently, more and more data suggest lncRNAs are mainly divided into two functional categories that enhance or repress the transcription or translation of protein-coding genes. Previous studies have described T-bet may regulate the expression of *Ifng* via inducing active transcription of *Ifng-AS1* (*Tmevpg1, NeST*), a Th1-specific lncRNA that contributes to the transcription of *Ifng*^25^,^26^. Transgenic mice models also have demonstrated that *Ifng-AS1(NeST)* plays a critical role in increasing Theiler’s virus persistence and resisting *Salmonella enterica* pathogenesis^30^. However, little is known regarding the role of *IFNG-AS1* in the disease pathogenesis of HT. Based on this, we speculated that the expression of *IFNG-AS1* might contribute to increased Th1 cells in HT patients. As expected, a higher transcript level of *IFNG-AS1* was found in peripheral blood and thyroid tissues from HT patients. Furthermore, positive correlations were found between the transcript level of *IFNG-AS1* and the proportion of Th1 cells, as well as transcript level of T-bet or *IFNG* in HT patients. Although a study has reported that *Tmevpg1* promoted the transcription of *Ifng* from murine Th1 cells *in vitro*^25^, the influence of *IFNG-AS1* on *IFNG* transcription in humans is still unknown. To investigate the role of *IFNG-AS1* in human CD4^+^ T cells, we used *IFNG-AS1*-specific siRNA to knockdown *IFNG-AS1* and observed the alteration of *IFNG*. *IFNG-AS1* knockdown resulted in a considerable reduction of *IFNG* in gene expression and protein expression (IFN-γ). Interestingly, *IFNG-AS1*-specific siRNA downregulated the proportion of CD4^+^ IFN-γ^+^ cells in a dose-dependent manner. In the present study, we provide direct evidence that *IFNG-AS1* regulates the transcription of *IFNG* from human Th1 cells *in vitro*. However, the epigenetic mechanism of *Ifng-AS1* promotes *Ifng* expression in Th1 cells is poorly understood.

The expression of *Tmevpg1* was found in Th1 cells from both mice and human, but was not detected in effector CD8^+^ T cells under the same culture conditions, which also produced IFN-γ^25^. Our supplemental data also demonstrated that there was no correlation between the transcript level of *IFNG-AS1* and increased proportion of CD8^+^ IFN-γ^+^ T cells in HT patients. One possible interpretation is the different mechanisms of IFN-γ production between Th1 cells and CD8^+^ T cells. T-bet is the master regulator of *Ifng* expression in Th1 cells^14^. However, IFN-γ production by effector CD8^+^ T cells is dependent of Eomesodermin (Eomes), a paralogue of T-bet, which plays key role in the differentiation and function of CD8^+^ T cells^31^. Another possible interpretation is the difference in regulating *IFNG* expression by *IFNG-AS1* between Th1 cells and CD8^+^ T cells. Although one model is generally indicated that *Ifng-AS1(NeST)* was required for *Ifng* expression in response to infection with *Salmonella*, and *Ifng-AS1(NeST)* was found to bind WDR5 component of histone H3 lysine 4 methytransferase complex, and to modify H3K4me3 at the *Ifng* locus by CD8^+^ T cells^30^. The differences between these reported findings may be due to different animal models, and the further mechanisms will be investigated in future.

Accumulating studies have demonstrated that HT is a Th1-mediated autoimmune disease because there are abundant Th1 cells infiltrating and thyrocyte destruction in HT patients^14^,^31^,^32^. Our results also showed that the proportion of Th1 cells and related genes, such as T-bet and *IFNG*, were higher in peripheral blood and thyroid gland from HT patients. It is widely accepted that elevated serum concentrations of TgAb and TPOAb are the most common manifestations of HT. These autoantibodies could indicate the development of HT, and IFN-γ production by Th1 cells drives the generation of autoantibodies^28^. We analyzed the correlation between the transcript level of *Ifng-AS1* and serum level of autoantibodies. Positive correlations were found between the transcript level of *Ifng-AS1* and the level of TgAb or TPOAb. These data suggest that *IFNG-AS1* expression could reflect disease severity of HT to some extent.

In summary, our results demonstrate that the lncRNA *IFNG-AS1* is significantly increased and may contribute to the pathogenic role of Th1 cells response in HT patients. Further exploration of the mechanism of *IFNG-AS1*-driven Th1 cells response may lead to better understanding of the pathogenesis of HT.

## Additional Information

**How to cite this article**: Peng, H. *et al.* The Long Noncoding RNA *IFNG-AS1* Promotes T Helper Type 1 Cells Response in Patients with Hashimoto's Thyroiditis. *Sci. Rep.* **5**, 17702; doi: 10.1038/srep17702 (2015).

## Figures and Tables

**Table 1:** Clinical features of the HT patients and healthy controls included in the study.

	HT patients	Healthy controls	Range	P-values
Number	28	20		
Gender (M/F)	5/23	3/17		
Age (year)	42 ± 15	42 ± 8		0.98
FT3 (pmol/liter)	4.87 ± 0.64	4.99 ± 0.56	3.1–6	0.51
FT4 (pmol/liter)	10.42 ± 2.36	10.72 ± 1.36	7.86–17.41	0.61
TSH (uIU/ml)	7.26 ± 11.26	1.98 ± 0.94	0.34–5.6	<0.05
TgAb (IU/ml)	162.6 ± 349.9	0.3 ± 1.0	0–4	<0.05
TPOAb (IU/ml)	570.3 ± 883.5	0.6 ± 0.5	0–9	<0.01

Data correspond to the arithmetic mean ± SD and were compared using unpaired t-tests. M: male; F: female.
